# Excision Depth Versus Margin Status After Large Loop Excision of the Transformation Zone (LLETZ): Which Predicts Cure?

**DOI:** 10.7759/cureus.109734

**Published:** 2026-05-27

**Authors:** Ei Mon Mon Kyaw, Mya Kyar Phyu

**Affiliations:** 1 Obstetrics and Gynaecology, Luton and Dunstable University Hospital NHS Foundation Trust, Luton, GBR

**Keywords:** cervical dysplasia, cervical intraepithelial neoplasia, cervical screening, cin3, colposcopy, excision depth, hrhpv, lletz, margin status, test of cure

## Abstract

Background

Large loop excision of the transformation zone (LLETZ) is the standard treatment for high-grade cervical intraepithelial neoplasia (CIN). Adequate excision depth and complete margin clearance are considered important for disease eradication; however, deeper excisions may increase obstetric morbidity. Identifying which surgical factors most strongly predict successful treatment is clinically important.

Methods

We conducted a retrospective cohort study of women undergoing LLETZ in our colposcopy unit. Data collected included age, referral cytology, excision depth, margin status, histology, and test of cure (high-risk human papillomavirus (hrHPV) and cytology). Successful treatment was defined as hrHPV negativity with no reflex cytology. Excision depth was categorised as ≤7 mm or ≥8 mm. Associations between depth, margin status, and outcomes were analysed using odds ratios (ORs).

Results

A total of 143 women who underwent LLETZ (mean age 39.6 years) were involved in this study. Most referrals were for high-grade cytology (76.9%). Histology confirmed CIN2+ in 65.0%, with occult invasive carcinoma and glandular neoplasia identified in 2.1% and 2.8%, respectively. The mean excision depth was 9.9 mm; 74.1% measured ≥8 mm. Clear margins were achieved in 67.8%, involved in 19.6%, and indeterminate in 12.6%.

Test-of-cure results were available for 121/143 (84.6%). Overall, 73.6% achieved hrHPV negativity. Deeper excisions showed modestly higher cure rates compared with shallow excisions (75.6% vs. 67.7%; OR 1.47). In contrast, margin status strongly predicted outcomes: cure was achieved in 85.4% with clear margins compared with 50.0% with involved margins, corresponding to nearly sixfold higher odds of failure with margin involvement (OR 5.8, 95% confidence interval (CI) 2.2-15.3). Most failures reflected isolated HPV persistence, while high-grade cytological recurrence was uncommon (4%).

Conclusion

Margin clearance is the principal determinant of successful treatment following LLETZ. Excision depth demonstrated only a modest association with cure, whereas involved margins markedly increased the risk of persistent disease. Optimising excision technique to achieve complete clearance, rather than simply increasing cone depth, may improve outcomes while minimising unnecessary cervical tissue loss.

## Introduction

Large loop excision of the transformation zone (LLETZ) is a standard excisional treatment for high-grade cervical intraepithelial neoplasia (CIN) and is central to cervical cancer prevention [1]. Excisional treatment aims to remove dysplastic epithelium completely while preserving cervical integrity and minimising treatment-related morbidity [1,2]. Current UK colposcopy guidance recommends adequate excision depth according to transformation zone type, while also recognising the importance of follow-up after treatment [1].

Excision depth and margin status are commonly used indicators of treatment adequacy following LLETZ. Incomplete excision, reflected by involved margins, has been associated with an increased risk of residual or recurrent high-grade disease [3,4]. However, increasing excision depth may also increase the risk of adverse obstetric outcomes, including preterm birth, particularly in women of reproductive age [2]. This creates an important clinical balance between achieving disease clearance and avoiding unnecessary cervical tissue removal.

High-risk human papillomavirus (hrHPV)-based test of cure is an established follow-up strategy after treatment for CIN, as persistent hrHPV positivity is an important marker of residual or recurrent disease [1,5]. Although excision depth and margin involvement have been studied individually, their relative contribution to successful test-of-cure outcomes remains clinically relevant. This study evaluated the relationship between excision depth, margin status, and test-of-cure outcomes in women undergoing LLETZ within a UK colposcopy service.

## Materials and methods

This retrospective cohort study was conducted within a UK colposcopy service and included women who underwent LLETZ for abnormal cervical cytology or biopsy-confirmed CIN. Consecutive patients undergoing LLETZ during the study period were identified from colposcopy records and electronic patient databases.

Data collected included patient age, referral cytology, excision depth, histological findings, excision margin status, and six-month test-of-cure outcomes. Referral cytology was categorised as high-grade, low-grade, borderline, or glandular neoplasia. Histological outcomes were grouped into high-grade disease (CIN2+), low-grade disease (CIN1), benign/negative findings, glandular neoplasia, and invasive carcinoma.

Excision depth was recorded in millimetres and categorised into shallow excisions (≤7 mm) and deeper excisions (≥8 mm). Margin status was classified as clear, positive, or indeterminate. Test of cure consisted of hrHPV testing with reflex cytology according to national cervical screening guidance. Successful treatment was defined as hrHPV negativity with no reflex cytology at follow-up. Unsuccessful outcomes included persistent hrHPV positivity with negative cytology, borderline or low-grade cytological abnormalities, and moderate/severe dyskaryosis or cervical glandular intraepithelial neoplasia (CGIN).

Descriptive statistics were used to summarise demographic, cytological, histological, and treatment characteristics. Associations between excision depth, margin status, and test-of-cure outcomes were assessed using odds ratios (ORs). Test-of-cure analyses were performed only in women with available follow-up data.

## Results

A total of 143 women underwent LLETZ during the study period. The mean age was 39.6 years. Most referrals were for high-grade cytology (110/143, 76.9%), followed by low-grade cytology (17/143, 11.9%), borderline cytology (12/143, 8.4%), and glandular neoplasia (4/143, 2.8%) (Table 1).

**Table 1:** Baseline Characteristics and Referral Cytology Referral cytology categories were based on pre-treatment cervical cytology findings.

Variable	Number (n)	Percentage
Total patients	143	
Mean age	39.6	
High grade	110	76.90%
Low grade	17	11.90%
Borderline	12	8.40%
Glandular neoplasia	4	2.80%
Total	143	100%

Histological outcomes following LLETZ are summarised in Table 2. High-grade disease (CIN2+) was identified in 93/143 (65.0%) specimens. Low-grade disease (CIN1) and benign or negative histology were identified in 15/143 (10.5%) and 28/143 (19.6%) cases, respectively. Glandular neoplasia and occult invasive carcinoma were identified in 4/143 (2.8%) and 3/143 (2.1%) cases, respectively.

**Table 2:** Histological Outcomes Following LLETZ (Large Loop Excision of the Transformation Zone) High-grade disease included cervical intraepithelial neoplasia grade 2 (CIN2), cervical intraepithelial neoplasia grade 3 (CIN3), and mixed CIN2/3 lesions. Glandular neoplasia included cervical glandular intraepithelial neoplasia (CGIN). Occult invasive carcinoma included International Federation of Gynaecology and Obstetrics (FIGO) stage IB1 squamous cell carcinoma (SCC) and stage IA1 cervical carcinoma.

Histology group	Number (n)	Percentage
High-grade (CIN2+)	93	65.0%
Low-grade (CIN1)	15	10.5%
Negative/benign	28	19.6%
Glandular (CGIN ± CIN)	4	2.8%
Invasive cancer	3	2.1%
Total	143	100%

Excision depth and margin status are presented in Table 3. The mean excision depth was 9.9 mm. Most excisions measured ≥8 mm (106/143, 74.1%), while 37/143 (25.9%) measured ≤7 mm. Clear margins were achieved in 97/143 (67.8%) cases, involved in 28/143 (19.6%), and indeterminate in 18/143 (12.6%).

**Table 3:** Excision Depth and Margin Status Large loop excision of the transformation zone (LLETZ) depth was categorised as ≤7 or ≥8 mm for comparative outcome analysis.

Variable	Number (n)	Percentage
Mean excision depth	9.9 mm	
Excision depth ≤ 7 mm	37	25.9%
Excision depth ≥ 8 mm	106	74.1%
Clear margins	97	67.8%
Positive margins	28	19.6%

Test-of-cure outcomes according to excision depth are shown in Table 4. Follow-up results were available for 121/143 women (84.6%), of whom 89/121 (73.6%) achieved successful test of cure with hrHPV negativity and no reflex cytology. Persistent hrHPV positivity with negative cytology was the most common cause of treatment failure (19/121, 15.7%), while borderline or low-grade cytological abnormalities and moderate/severe dyskaryosis or CGIN accounted for 8/121 (6.6%) and 5/121 (4.1%) cases, respectively. Women undergoing deeper excisions (≥8 mm) demonstrated modestly higher cure rates compared with those undergoing shallow excisions (75.6% vs. 67.7%), corresponding to an OR of 1.47 for treatment failure with shallow excisions (Table 4).

**Table 4:** Test-of-Cure (TOC) Outcomes by Excision Depth Successful TOC was defined as high-risk human papillomavirus (hrHPV) negativity with no reflex cytology.

Excision depth	Successful TOC n (%)	Failed TOC n (%)	Total	Cure rate
≤7 mm	21 (67.7%)	10 (32.3%)	31	67.7%
≥8 mm	68 (75.6%)	22 (24.4%)	90	75.6%
Total	89 (73.6%)	32 (26.4%)	121	73.6%

Test-of-cure outcomes according to margin status are presented in Table 5. Women with clear margins achieved substantially higher cure rates than those with involved margins (85.4% vs. 50.0%). Margin involvement was associated with nearly sixfold higher odds of treatment failure (OR 5.8, 95% confidence interval 2.2-15.3). Women with indeterminate margins also demonstrated lower cure rates (46.7%) (Table 5).

**Table 5:** TOC Outcomes by Margin Status Margin involvement was associated with significantly lower cure rates following LLETZ. TOC: test of cure; hrHPV: high-risk human papillomavirus; LLETZ: large loop excision of the transformation zone

Margin status	Successful TOC n (%)	Failed TOC n (%)	Total	Cure rate
Clear	70 (85.4%)	12 (14.6%)	82	85.4%
Positive	12 (50.0%)	12 (50.0%)	24	50.0%
Uncertain	7 (46.7%)	8 (53.3%)	15	46.7%
Total	89 (73.6%)	32 (26.4%)	121	73.6%

## Discussion

This study evaluated the relative impact of excision depth and margin status on test-of-cure outcomes following LLETZ within a UK colposcopy service. Our findings demonstrated that margin status was a substantially stronger predictor of treatment success than excision depth. Women with clear excision margins achieved cure rates exceeding 85%, whereas involved margins were associated with almost sixfold higher odds of treatment failure. In contrast, deeper excisions demonstrated only a modest improvement in cure rates compared with shallow excisions.

Histological analysis confirmed high-grade CIN (CIN2+) in 65% of excised specimens, supporting appropriate patient selection within a predominantly high-grade referral population. The overall rate of involved margins in our cohort (19.6%) is comparable with previously reported rates following LLETZ, which generally range between 15% and 25% [3,4]. Margin involvement has consistently been identified as a major predictor of residual or recurrent disease following excisional treatment [3,4]. Arbyn et al. demonstrated significantly increased risk of recurrent CIN2+ in women with involved margins following treatment for cervical precancer [4]. Similarly, Ghaem-Maghami et al. reported markedly higher recurrence rates in women with incomplete excision compared with those with clear margins [3].

Our findings strongly support these observations. Women with involved margins in our study achieved substantially lower cure rates than those with clear margins (50.0% vs. 85.4%). Importantly, the OR for treatment failure associated with margin involvement (OR 5.8) was considerably greater than that observed for shallow excision depth (OR 1.47). This suggests that excision quality, reflected by complete disease clearance, is more clinically important than excision depth alone. These findings are also consistent with evidence demonstrating that negative hrHPV testing combined with clear margins provides strong reassurance against recurrent CIN2+ [5].

Although deeper excisions demonstrated modestly improved cure rates in our cohort, the benefit appeared limited when compared with the effect of margin clearance. This observation is clinically important because increasing excision depth has been associated with adverse obstetric outcomes, particularly preterm birth [2,6-8]. Kyrgiou et al. demonstrated a depth-dependent increase in obstetric morbidity following cervical excisional treatment [2,6], while Castanon et al. reported increased risk of preterm delivery with increasing depth of cervical excision [7]. Noehr et al. similarly demonstrated an association between larger cone depth and subsequent spontaneous preterm birth [8]. Our findings therefore support a balanced surgical approach aimed at achieving complete excision while avoiding unnecessary removal of cervical tissue.

Previous studies have also attempted to define optimal excision dimensions for achieving adequate disease clearance while minimising cervical morbidity. Papoutsis et al. suggested that appropriate cone dimensions may improve the likelihood of negative excision margins while reducing unnecessary tissue excision [9]. Our findings support this principle, as deeper excisions demonstrated only modest improvement in cure rates when compared with the much stronger effect of margin clearance.

The majority of treatment failures in our study reflected isolated persistent hrHPV positivity with negative cytology rather than recurrent high-grade disease. High-grade cytological recurrence occurred in only 4% of women with follow-up data, suggesting overall effective treatment outcomes within the service. Persistent hrHPV positivity following treatment is recognised as an important predictor of residual disease and underpins current test-of-cure strategies [1,5].

A small number of women in our cohort were diagnosed with glandular neoplasia or occult invasive carcinoma on final histology. Margin involvement was particularly common in glandular lesions, reflecting the recognised difficulty in achieving complete excision in CGIN. Careful follow-up remains important in women with glandular disease.

The strengths of this study include a detailed assessment of excision depth, margin status, histological outcomes, and test-of-cure results within routine clinical practice. However, the study is limited by its retrospective design and incomplete follow-up in a proportion of women. In addition, margin status was analysed collectively without differentiating endocervical from ectocervical involvement, which may have differing prognostic significance. Furthermore, important clinical variables including smoking status, immunosuppression, contraceptive use, and lesion size were not evaluated, which may have influenced recurrence risk and test-of-cure outcomes following LLETZ.

Overall, our findings suggest that clear excision margins are the key determinant of successful treatment following LLETZ. While deeper excisions showed only a modest association with improved cure rates, involved margins were strongly associated with persistent disease. These findings support optimising the excision technique to achieve complete disease clearance while minimising unnecessary cervical tissue removal and potential reproductive morbidity.

## Conclusions

Clear excision margins were strongly associated with successful test-of-cure outcomes following LLETZ, whereas excision depth demonstrated only a modest association with treatment success. Women with involved margins had substantially higher rates of persistent hrHPV infection and abnormal follow-up cytology compared with those with clear margins. These findings suggest that achieving complete disease clearance is more important than increasing cone depth alone. Optimising excision technique to achieve adequate margins while minimising unnecessary cervical tissue removal may improve treatment outcomes and reduce potential reproductive morbidity.
